# New Oxicam Derivatives—Studies of Membrane Interactions, Cytotoxicity, Cyclooxygenase Inhibition and Molecular Docking

**DOI:** 10.3390/membranes16050166

**Published:** 2026-05-01

**Authors:** Jadwiga Maniewska, Katarzyna Gębczak, Łucja Cwynar-Zając, Żaneta Czyżnikowska, Berenika M. Szczęśniak-Sięga

**Affiliations:** 1 Department of Medicinal Chemistry, Faculty of Pharmacy, Wroclaw Medical University, Borowska 211, 50-556 Wroclaw, Poland; 2 Division of Basic Medical Sciences, Department of Basic Medical Sciences and Immunology, Faculty of Pharmacy, Wroclaw Medical University, Borowska 211, 50-556 Wroclaw, Poland; 3 Department of Basic Chemical Sciences, Faculty of Pharmacy, Wroclaw Medical University, Borowska 211a, 50-556 Wroclaw, Poland

**Keywords:** model membranes, differential scanning calorimetry (DSC), oxicam derivatives, cyclooxygenase, ROS, molecular docking

## Abstract

Oxicam derivatives, a class of nonsteroidal anti-inflammatory drugs (NSAIDs), are important scaffolds for developing biologically active compounds. In this study, arylpiperazine oxicam derivatives (PR24–PR50) were examined for membrane interactions, cytotoxic activity, cyclooxygenase inhibition, and potential binding to COX-2 protein. Membrane interactions were examined using differential scanning calorimetry (DSC) in phospholipid bilayers formed from 1,2-dimyristoyl-*sn*-glycero-3-phosphocholine (DMPC). All compounds altered the thermotropic properties of the lipid bilayer, showing concentration-dependent decreases in phase transition temperature, indicating incorporation to bilayer and partial disruption of lipid organization. Cytotoxicity, assessed using the MTT assay in breast cancer (MCF-7, MCF-7/DX), colorectal cancer (LOVO, LOVO/DX), and normal V79 cell lines, showed moderate effects, particularly against colorectal cancer cells. Cyclooxygenase inhibition was rather weak, with IC_50_ values in the high micromolar range, indicating limited anti-inflammatory potential compared with reference COX inhibitors, although docking studies suggested possible interactions with the COX-2 active site. The obtained results indicate that the biological activity of the arylpiperazine oxicam derivatives is primarily associated with cytotoxicity and membrane effects rather than COX inhibition. These limitations should be considered in the design of future membrane-targeted bioactive compounds.

## 1. Introduction

Oxicams are a subgroup of nonsteroidal anti-inflammatory drugs (NSAIDs) known for their ability to non-selectively inhibit cyclooxygenase 1 and 2 (COX-1 and COX-2) [1]. Since the discovery that inflammatory processes are involved in carcinogenesis, anti-inflammatory drugs have become a target of research to support many anticancer therapies [2]. Cancer is still a leading cause of death worldwide—ranking as the first or second cause of death before the age of 70 in 112 out of 183 countries [3]. The pathophysiology of carcinogenesis is very complex and still not fully understood. Cancer is a pathological condition in which homeostasis in cell proliferation and differentiation is disturbed [4,5]. Less than 10% of cancers are caused by germline mutations, with most cases arising from somatic changes and environmental factors. Cancer is often associated with diet, obesity, inhaled pollutants, smoking, or an autoimmune reaction [6,7,8]. Moreover, chronic inflammation is recognized as a key pathological factor, representing a prolonged and dysregulated response to the loss of tissue homeostasis [9]. Cancer development may be viewed as a dysregulated repair response to tissue damage, where inflammatory and neoplastic processes co-exist, forming the concept of a “wound that does not heal” [10].

The link between inflammation and tumor development was first observed in 1863 by Rudolf Virchow, founder of modern pathology [11]. Virchow observed tumors in several patients where they had been struck or repeatedly irritated by shoes or work tools. Examining the tumor tissues under a microscope, he observed leukocyte infiltration. He hypothesized that cancer resulted from a failed attempt by the body’s immune system to repair the wound. However, scientists did not take this concept seriously at the time. Only more than a century later, in 1986, Harold Dvorak, a pathologist at Harvard University, demonstrated a surprising similarity between the mechanisms naturally occurring in the inflammatory process and the development of tumor tissue [10]. Dvorak also noted that cancer is often directly related to chronic inflammation, a finding confirmed by the other subsequent research [12]. Inflammation arising from chronic infections is estimated to contribute to around 25% of the worldwide cancer burden [13]. Furthermore, chronic inflammatory processes triggered by chemical and physical agents, autoimmune factors, as well as reactions of unknown origin, are linked to an increased cancer risk [14].

Additionally, the use of NSAIDs for analgesic and anti-inflammatory purposes has revealed their chemopreventive effects in several cancers, including breast, lung, bladder, colon, and prostate cancers [15,16,17,18,19,20,21,22,23,24,25,26,27,28,29,30,31]. NSAIDs inhibit cyclooxygenase, an enzyme with at least two isoforms, COX-1 and COX-2. COX-1 is a constitutively expressed glycoprotein present in various tissues, including the stomach, intestines, ovaries, kidneys, and platelets, with important protective functions [32]. COX-2 is an inducible isoform upregulated by cytokines (IL-1β, IL-6, TNFα), mitogens, and growth factors during inflammation, pain, tissue damage, and carcinogenesis. Its overexpression, linked to increased prostaglandin E_2_ (PGE_2_) production, plays a key role in carcinogenesis and chronic inflammation [33]. COX-2 has been shown to participate in cancer development at multiple stages, from tumor initiation to metastasis [34]. The protumorigenic activity of COX-2 is thought to involve several mechanisms, including stimulation of mutant cell proliferation, inhibition of apoptosis, reduced epithelial cell adhesion, promotion of angiogenesis, increased invasiveness, modulation of immune surveillance, induction of DNA mutagenesis, and activation of carcinogenic xenobiotics through peroxidase activity [34].

Over the last three decades, numerous reports have emerged from experimental animal studies, epidemiological studies, and clinical trials showing that regular use of aspirin and other NSAIDs, including celecoxib, piroxicam, and sulindac, has been associated with a reduction in cancer risk of up to 50% [35]. Some anti-inflammatory drugs have even been approved for use in cancer treatment or as chemopreventive agents; for example, celecoxib is used to reduce the risk of colorectal adenomas in people with familial adenomatous polyposis [36]. Oxicams, such as piroxicam or meloxicam, are characterized by potent anti-inflammatory activity and a long duration of action, and were therefore also targeted for such studies. However, piroxicam was withdrawn from further studies due to its significant toxicity. To reduce its toxicity, new arylpiperazine oxicam derivatives were designed and synthesized. Some of them demonstrated anticancer or potential chemopreventive properties [37,38,39,40,41].

A series of novel arylpiperazine oxicam derivatives (Table 1) was investigated for anti-inflammatory and cytotoxic properties. The anti-inflammatory potential was evaluated in vitro by measuring inhibition of COX-2, an enzyme involved in both inflammatory and pro-carcinogenic processes, whereas COX-1 inhibition was additionally assessed to define the COX-2/COX-1 selectivity profile. Due to the fact that cyclooxygenase is an enzyme anchored in the endoplasmic reticulum, drug permeation across biological membranes is necessary for interaction with the enzyme. For this purpose, studies of the interaction of oxicam derivatives with model biological membranes were also conducted. In addition, molecular docking studies focused on the COX-2 active site were conducted to investigate the binding mode of the compounds. The cytotoxic activity of the new oxicam derivatives was subsequently tested in two cancer cell lines, along with their doxorubicin-sensitive and doxorubicin-resistant sublines. What is more, to determine the compounds’ safety, cytotoxicity was also tested on a normal cell line. The mechanism of cytotoxic action was determined by testing the compounds’ activity on rhodamine-containing cells. In addition, the antioxidant activity of the new compounds was evaluated, as inflammation is associated with the generation of reactive oxygen and nitrogen species (ROS and RNS). These species may damage essential cellular components, including DNA, proteins, and lipids, thereby contributing—directly or indirectly—to neoplastic transformation.

## 2. Materials and Methods

### 2.1. Chemicals

Oxicam derivatives were obtained through synthesis performed at the Department of Medicinal Chemistry, Wroclaw Medical University, Poland. Chemical structures, molecular weights and abbreviations of studied oxicam derivatives are shown in Table 1. The synthesis route and spectral spectra confirming structures and purity of new compounds have been published previously [42,43]. Meloxicam and celecoxib (reference standards) were supplied by Thermo Fisher Scientific (Waltham, MA, USA). Given the lack of water solubility of the investigated compounds, they were dissolved in chloroform or dimethyl sulfoxide (DMSO) prior to use in the assays.

### 2.2. Differential Scanning Calorimetry (DSC)

The thermal response of phospholipid bilayers in the presence of oxicam derivatives was examined using 1,2-dimyristoyl-*sn*-glycero-3-phosphocholine (DMPC; Sigma-Aldrich, St. Louis, MO, USA) as a representative membrane lipid. The investigated compounds were initially dissolved in chloroform to obtain 5 mM stock solutions. Defined amounts of these solutions were subsequently combined with DMPC (2 mg per sample) to yield compound-to-lipid molar fractions ranging from 0.02 to 0.1. Following mixing, organic solvent was removed by evaporation under a stream of nitrogen, and samples were further subjected to vacuum drying for 2 h to eliminate residual traces. The resulting lipid films were rehydrated with 20 µL of Tris–EDTA buffer (20 mM, pH 7.4; Sigma-Aldrich), then heated above the main phase transition temperature of DMPC and vortexed to produce uniform dispersions. Prepared samples were hermetically sealed in aluminum *Concavus pans* (Netzsch GmbH & Co., Selb, Germany) and analyzed by differential scanning calorimetry using a DSC 214 *Polyma* instrument (Netzsch GmbH & Co., Selb, Germany) operating in heat-flow mode. Measurements were conducted over the temperature range of 10–35 °C at a constant heating rate of 1 °C/min under a nitrogen purge (25 mL/min). For each composition, two independently prepared samples were measured, each in duplicate. Transition enthalpy values (J/g) were calculated with respect to lipid mass using Netzsch Proteus software (version 7.1.0). The experimental protocol was based on our previously reported methodology [44].

### 2.3. Biological Assays

The biological activity of the investigated compounds was assessed using four complementary assays: MTT, cyclooxygenase (COX) inhibition, DCFH-DA, and Rhodamine 123. These assays were employed to evaluate cell viability, COX inhibitory activity, intracellular reactive oxygen species (ROS) generation, and mitochondrial membrane potential (ΔΨm), respectively. Tests were performed for three cell lines: the normal V79 line, the breast cancer line MCF-7, and its doxorubicin-resistant variant MCF-7/DX, as well as the colorectal cancer line LOVO, and its resistant variant LOVO/DX. The V79 cell line is derived from the lung tissue of a young male Chinese hamster and displays fibroblast-like morphology [45]. The LOVO cell line originates from a metastatic lesion in the left supraclavicular region of a 56-year-old male patient with colorectal adenocarcinoma and is characterized by epithelial morphology [46]. The MCF-7 cell line was established in 1970 from pleural effusion obtained from a 69-year-old female patient with metastatic breast adenocarcinoma and is characterized by epithelial morphology [47]. The LOVO/DX and MCF-7/DX cell lines were created by cultivating parental counterparts in the presence of low concentrations of doxorubicin. Cells were seeded in 96-well plates at a density of 1–2 × 10^4^ cells per well and cultured for 24 h under standard conditions (37 °C, 5% CO_2_, humidified atmosphere) to allow cell adhesion. In all four assays, the cells were treated with the tested compounds at the same concentrations of 1, 10, and 50 µM. The starting concentration was chosen as 1 µM because doxorubicin, the reference drug, is often tested at this concentration, which facilitates comparison of the efficacy of the tested compounds. The subsequent concentrations—10 and 50 µM—were chosen after preliminary studies that demonstrated differences in the effects of the compounds between these concentrations. All measurements were performed using a Varioskan Microplate Reader (Thermo Fisher Scientific, Waltham, MA, USA).

#### 2.3.1. Determination of Cell Proliferation (MTT Assay)

Cell viability was assessed using the MTT assay based on the reduction of tetrazolium salt to formazan by metabolically active cells, as previously described, with minor modifications [48]. Following 24 h exposure to the tested compounds, MTT solution (1 mg/mL) was added to each well, and the cells were incubated for 2 h at 37 °C. Subsequently, the medium was removed, and the formed formazan crystals were dissolved in dimethyl sulfoxide (DMSO). Absorbance was measured at 570 nm, and cell viability was calculated as a percentage relative to untreated control cells (100%) [49]. Non-viable cells do not reduce MTT and therefore do not generate a detectable signal.

#### 2.3.2. Cyclooxygenase Inhibitory Activity

Cyclooxygenase (COX) inhibitory activity was determined using a colorimetric assay kit (Cayman Chemical Company, Ann Arbor, MI, USA) according to the manufacturer’s instructions [50]. The assay is a high-throughput microplate-based method that measures COX peroxidase activity via colorimetric detection of oxidized *N,N,N′,N′*-tetramethyl-*p*-phenylenediamine (TMPD) at 590 nm, enabling assessment of compounds that inhibit COX activity in vitro. The assay was performed according to the manufacturer’s protocol using 0.1 M Tris-HCl buffer (pH 8.0), heme solution in DMSO, COX-1 or COX-2 enzymes, arachidonic acid (100 µM), KOH (0.1 M), and TMPD solution. The reaction mixture consisted of 150 µL assay buffer, 10 µL heme, and 10 µL of COX-1 or COX-2 enzyme per well. Test samples were prepared by adding 10 µL of the investigated compounds at final concentrations of 1, 10, and 50 µM. For control wells, 10 µL of DMSO was used instead of the compound. The reaction was initiated by the addition of 20 µL TMPD, followed by the addition of arachidonic acid, and allowed to proceed for 2 min at 25 °C. The oxidation of TMPD was measured using a Varioskan Lux microplate reader (Thermo Scientific, USA) at 590 nm. Inhibitory activity was expressed as the concentration required to inhibit enzyme activity by 50% (IC_50_). Selectivity was assessed using the selectivity index (SI), calculated as IC_50_ (COX-2)/IC_50_ (COX-1).

#### 2.3.3. Reactive Oxygen Species Assay

Intracellular reactive oxygen species (ROS) levels were determined using the DCFH-DA assay (Sigma-Aldrich, St. Louis, MO, USA) as described elsewhere [48,51]. Following 24 h exposure to the tested compounds (1, 10, and 50 µM), the culture medium was removed and cells were washed with phosphate-buffered saline (PBS). Subsequently, DCFH-DA solution (20 µM in PBS) was added, and the cells were incubated for 30 min at 37 °C in the dark. After incubation, cells were washed with PBS, and fluorescence of the oxidized product (DCF) was measured using excitation and emission wavelengths of 485 nm and 530 nm, respectively. Results were expressed as a percentage of the untreated control (100%), with increased fluorescence corresponding to elevated ROS production.

#### 2.3.4. Rhodamine 123 Assay

Mitochondrial membrane potential was evaluated using the Rhodamine 123 assay (Sigma-Aldrich, St. Louis, MO, USA) following a previously reported procedure [52]. After 24 h exposure to the tested compounds (1, 10, and 50 µM), the culture medium was removed and cells were washed with phosphate-buffered saline (PBS). Cells were then incubated with Rhodamine 123 solution (5 µM in PBS) for 30 min at 37 °C in the dark. Following incubation, cells were washed with PBS, and fluorescence intensity was measured at excitation and emission wavelengths of 505 nm and 530 nm, respectively [53]. Results were expressed as a percentage of control fluorescence (100%), where a decrease in signal indicated loss of mitochondrial membrane potential.

#### 2.3.5. Statistical Analysis

Statistical analysis was performed using Statistica 13.3 software (TIBCO Software Inc., Palo Alto, CA, USA). Data distribution normality was evaluated using the Shapiro–Wilk test. For normally distributed data, comparisons between independent groups were performed using Student’s *t*-test. In cases where the assumption of normality was not satisfied, the nonparametric Mann–Whitney U test was applied [48,52]. Results are presented as mean ± standard deviation (SD). Statistical significance was defined as *p* < 0.05 (*), *p* < 0.01 (**), and *p* < 0.001 (***).

### 2.4. Computational Studies

The geometry of oxicam derivatives was optimized at the B3LYP/6-31++G** level of theory using the polarizable continuum model (PCM) to include the solvent effects. The calculations were performed by using the Gaussian 09 program [54,55,56,57,58]. The standard AutoDock 4.2 protocol was employed to obtain the binding modes of the compounds within the COX-2 binding cavity [59]. The crystal structure of cyclooxygenase-2 (COX-2; PDB ID: 4M11), co-crystallized with meloxicam, was retrieved from the Protein Data Bank [60]. The structure of the molecular target (chain A) was prepared by adding polar hydrogen atoms and assigning appropriate solvation parameters. Gasteiger partial charges were assigned to all atoms. The binding site was defined using a grid box of 60 × 60 × 60 points centered on the active site based on the position of the co-crystallized ligand, with a grid spacing of 0.375 Å. Visualization was performed using Discovery Studio Visualizer 2021 (Dassault Systèmes, Waltham, MA, USA; https://discover.3ds.com/, accessed on 1 October 2025).

## 3. Results

### 3.1. Differential Scanning Calorimetry

**Figure 1:**
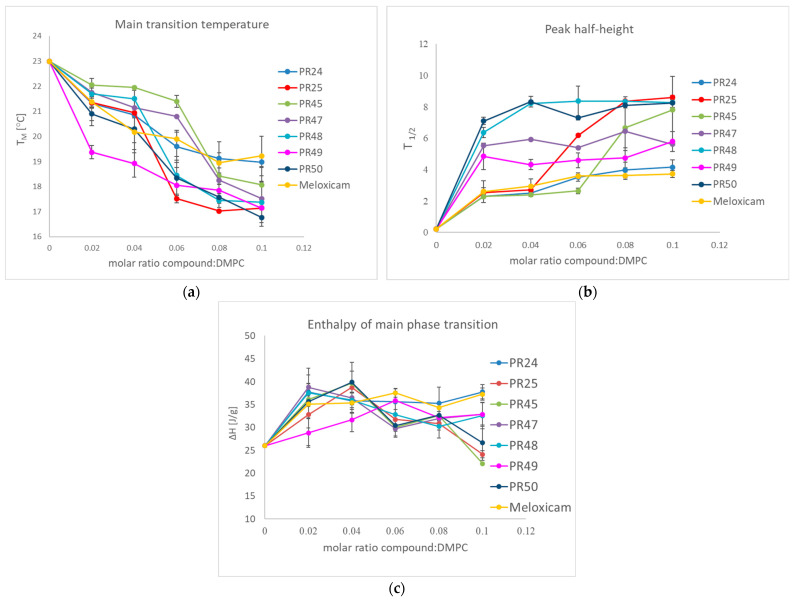
Influence of studied compounds on the DMPC: (**a**) main phase transition temperature; (**b**) main phase transition peak half-height width; (**c**) main phase transition enthalpy; mean ± SD, *n* = 4.

Differential Scanning Calorimetry (DSC) is one of the techniques used in studying drug–membrane interactions by measurements of the thermotropic phase behavior of phospholipids building the membrane [61]. In our research, this tool was used to characterize the influence of oxicam derivatives on model phospholipid membranes formed from DMPC, because this technique makes it possible to follow the effects of any additives to model membrane on cooperativity and enthalpy of the lipid phase transition. The dependencies of the main transition temperature, half-height width and enthalpy change on the oxicam derivative concentration are shown in Figure 1.

The investigated compounds had previously been studied on model biological membranes obtained from dipalmitoylphosphatidylcholine (DPPC) [42,43]. Herein they were examined using DMPC, a phospholipid characterized by shorter hydrocarbon chains than DPPC. Uniform molar ratios were applied to allow direct comparison of the obtained results. All tested compounds induced a concentration-dependent decrease in the main phase transition temperature of DMPC, indicating a destabilization of the ordered gel phase. In parallel, incorporation of the compounds into the phospholipid bilayer resulted in a broadening of the phase transition peaks, suggesting increased heterogeneity of the lipid environment and reduced cooperativity of the phase transition. Furthermore, a decrease in the enthalpy of the main DMPC phase transition was observed for all compounds, reflecting weakened lipid–lipid interactions. These findings demonstrate that the examined compounds interact with the phosphatidylcholine model membrane and significantly modify its thermotropic properties. The most pronounced effects on the transition temperature were observed for compounds PR25, PR49, and PR50. The nature of the observed changes suggests that oxicam derivatives affect both the polar headgroup region and the hydrocarbon chains of DMPC, leading to a reduction in the stability of the gel phase and weakened intermolecular interactions within the lipid bilayer [62].

### 3.2. Biological Assays

#### 3.2.1. Determination of Cell Proliferation

**Figure 2:**
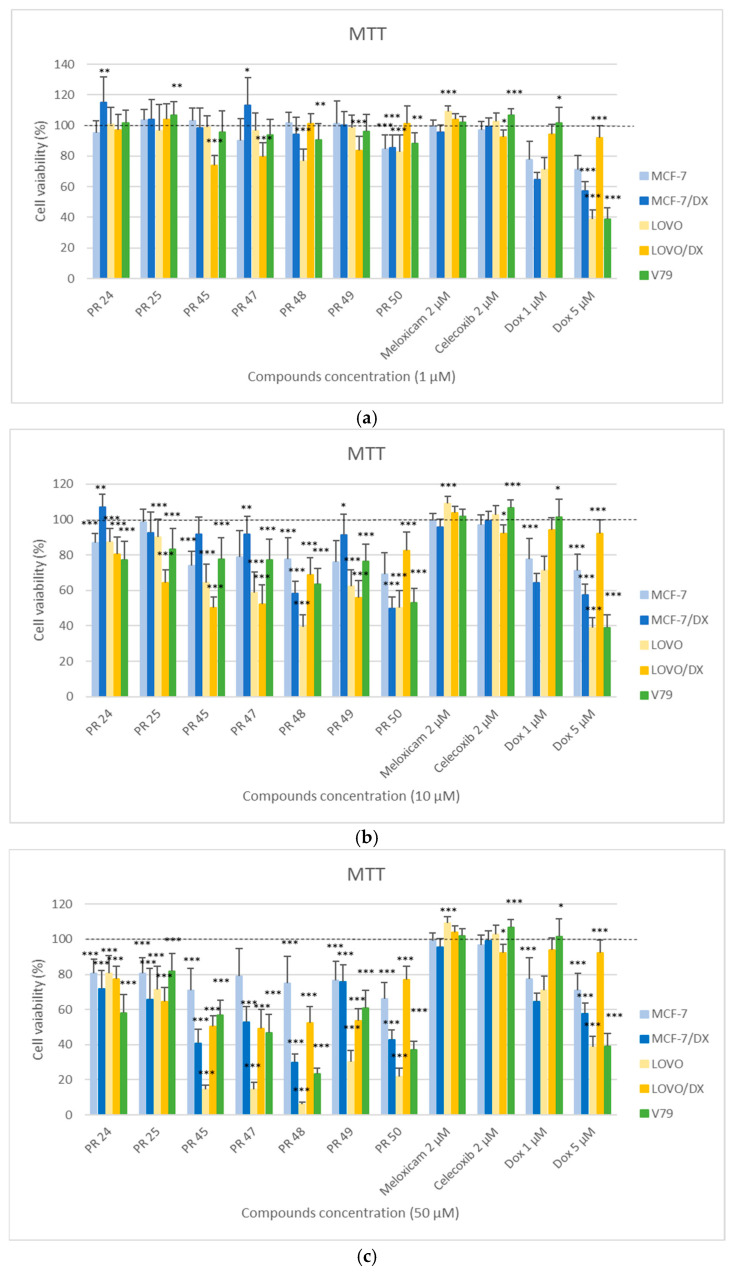
Effect of the tested compounds PR24–PR50 on the viability of cancer (MCF-7, MCF-7/DX, LOVO, LOVO/DX) and normal (V79) cells at a concentration of 1 µM (**a**), 10 µM (**b**), 50 µM (**c**), evaluated by the MTT assay after 24 h of incubation. Results are presented as the percentage of cell viability relative to the untreated control (100%). The presence of statistical significance markers (* *p* ≤ 0.05; ** *p* < 0.01; *** *p* < 0.001) indicates significant differences compared to the control group.

In Figure 2, the results of the MTT assay are presented, determining the effect of the tested compounds PR24–PR50 on the viability of cancer cells (MCF-7, MCF-7/DX, LOVO, LOVO/DX) and normal cells (V79) after 24 h of incubation at three concentrations: 1, 10, and 50 µM. Normal cell line V79 is a Chinese hamster lung fibroblast cell commonly used in toxicology studies. Cancer cell line MCF-7 is a breast cancer cell, and LOVO is a colorectal cancer cell. Cancer cell lines MCF-7/DX and LOVO/DX are their doxorubicin-resistant variants.

The MTT assay showed differentiated cytotoxicity of the tested derivatives depending on the cell type, the applied concentration, as well as the structure of tested compound. With increasing concentration, a clear decrease in cell viability was observed, confirming the dose-dependent nature of cytotoxicity. The strongest cytotoxic effect occurred at the concentration of 50 µM (Figure 2c), especially against the cancer cells LOVO and LOVO/DX (colon cancer cell lines). For most compounds (PR45, PR47, PR48, PR49, PR50), the viability of LOVO cells decreased below 40–60% compared to the control (100%). At the same time, the resistant LOVO/DX cells were characterized by higher viability, indicating persistent resistance to the tested derivatives.

In the breast cancer cell lines MCF-7 and MCF-7/DX, moderate cytotoxicity of most derivatives was observed. The most active compounds were again PR48 and PR50, causing a reduction in the viability of MCF-7/DX cells to about 60–70% at 50 µM. The MCF-7 (non-resistant) cells generally showed lower sensitivity to the tested compounds than LOVO cells, which may result from metabolic differences between cancer types.

In the case of normal V79 cells, the effect of the tested derivatives was varied and depended on the type of compound. Some derivatives, such as PR47 and PR50, showed moderate cytotoxicity. The lowest toxicity toward normal cells was exhibited by compound PR25, while PR48 showed the strongest effect in this line.

At lower concentrations of 1 and 10 µM (Figure 2a,b), most compounds showed a smaller effect on cell viability, although statistically significant results were obtained in most cases. It is worth emphasizing that compounds PR48 and PR50, which were most cytotoxic to cancer cells, were equally toxic to healthy cells. Compounds PR25, PR45, PR47, and PR49, on the other hand, were more cytotoxic to resistant colon cancer cells (LOVO/DX) than to healthy cells (V79).

The IC_50_ values (µM) for the tested derivatives PR24–PR50 were determined based on the results of the MTT assay using the linear regression method (y = ax + b). Calculations were performed for five cell lines: the normal cell line (V79), and two cancer lines (MCF-7, MCF-7/DX, LOVO, LOVO/DX). The results are presented in Table 2. The symbol nd (not determined) indicates that the IC_50_ value could not be determined within the tested concentration range.

The analysis of the obtained IC_50_ values confirmed the variable sensitivity of the tested cell lines to the studied compounds. The greatest cytotoxic activity against cancer cells was demonstrated by derivatives PR48 and PR50, which showed the lowest IC_50_ values—4.7 µM and 8.8 µM, respectively, for LOVO cells. High activity of these compounds was also confirmed in the resistant lines LOVO/DX (PR48—54.2 µM) and MCF-7/DX (PR48—15.3 µM and PR50—18.3 µM). Unfortunately, these compounds were also the most toxic to healthy cells V79 (PR48—13.7 µM and PR50—16.2 µM).

Compounds PR45, PR47 and PR49 exhibited moderate cytotoxicity toward cancer cell lines, with particularly high activity against LOVO cells (IC_50_ values of 12.3, 11.0, and 17.5 µM, respectively) and their resistant variant LOVO/DX (IC_50_ values of 28.7, 28.5, and 47.3 µM, respectively). However, against healthy cells V79 they showed very low toxicity (IC_50_ values of 123.0, 55.6, and 178.0 µM, respectively) which indicates their greater selectivity towards cancer cells.

In the case of the MCF-7 cell line, it was not possible to determine IC_50_ values for any of the tested compounds. This was due to the fact that cell viability across the entire tested concentration range (1–50 µM) remained at approximately 80–90%, which prevented the establishment of a dose–response relationship required to calculate IC_50_ values using the linear regression method. The obtained results indicate that MCF-7 cells exhibited low sensitivity to the tested derivatives.

A comparison of IC_50_ values between sensitive and resistant colon cancer cell lines showed that the resistant cells (LOVO/DX) generally exhibited higher IC_50_ values, suggesting reduced sensitivity to the tested compounds.

#### 3.2.2. Structure–Activity Relationship (SAR) Analysis

The two most cytotoxic compounds, both against cancer and healthy cell lines, PR48 and PR50, differ from the moderately active compounds PR25, PR45, PR47, and PR49 in the type of linker between the thiazine and piperazine nitrogen atoms. The former possesses a propylene linker, while the latter possesses an acetyl linker, indicating the importance of this linker not only for the compound’s overall toxicity but also for its activity profile. Compounds PR48 and PR50 also possess an *m*-trifluoromethylphenyl substituent on the piperazine moiety. The most cytotoxic compound, PR48, also has a bromine atom in its structure, while the less cytotoxic PR50 has a methyl group instead of bromine. This indicates that both the bromine atom and the *m*-trifluoromethyl substituent significantly enhance the compound’s cytotoxicity.

On the other hand, compounds with the lowest cytotoxicity are PR24 and PR25. Structurally, they are distinguished by the presence of *o*-fluorophenyl substituent on the piperazine. The remaining compounds tested possess *m*-trifluoromethyl substituent, demonstrating that this substituent significantly enhances their cytotoxicity. The three compounds that demonstrated the highest cytotoxicity against LOVO cell line were PR45, PR47, and PR48. In addition to *m*-trifluoromethyl substituent mentioned above, these compounds also contain chlorine atom (PR45) or bromine atom (PR47 and PR48), suggesting that these substituents affect cytotoxicity more selectively. Due to the limited number of compounds, it is not possible to draw more detailed conclusions on the structure–activity relationship.

#### 3.2.3. Cyclooxygenase Inhibitory Activity

The inhibitor concentration causing 50% inhibition of COX-1 and COX-2 activity (IC_50_) was calculated based on the experimental data for the tested compounds at three concentrations: 1 µM, 10 µM, 50 µM and is presented in Table 3. The selectivity index (SI) was calculated as the quotient of IC_50_ COX-2/IC_50_ COX-1.

All tested compounds showed much lower effect on COX-1 and COX-2 activity compared to the reference compound, celecoxib. Compounds PR24, PR45, PR47, and PR48 exhibited a detectable reduction in COX-1 and COX-2 activity only at higher concentrations than celecoxib, with a somewhat stronger reduction in COX-1 activity than COX-2. Compound PR49 affected only COX-2 activity. Compounds PR25 and PR50 showed minimal or no detectable effect on either COX-1 or COX-2 within the tested concentration range.

#### 3.2.4. Reactive Oxygen Species Assay

**Figure 3:**
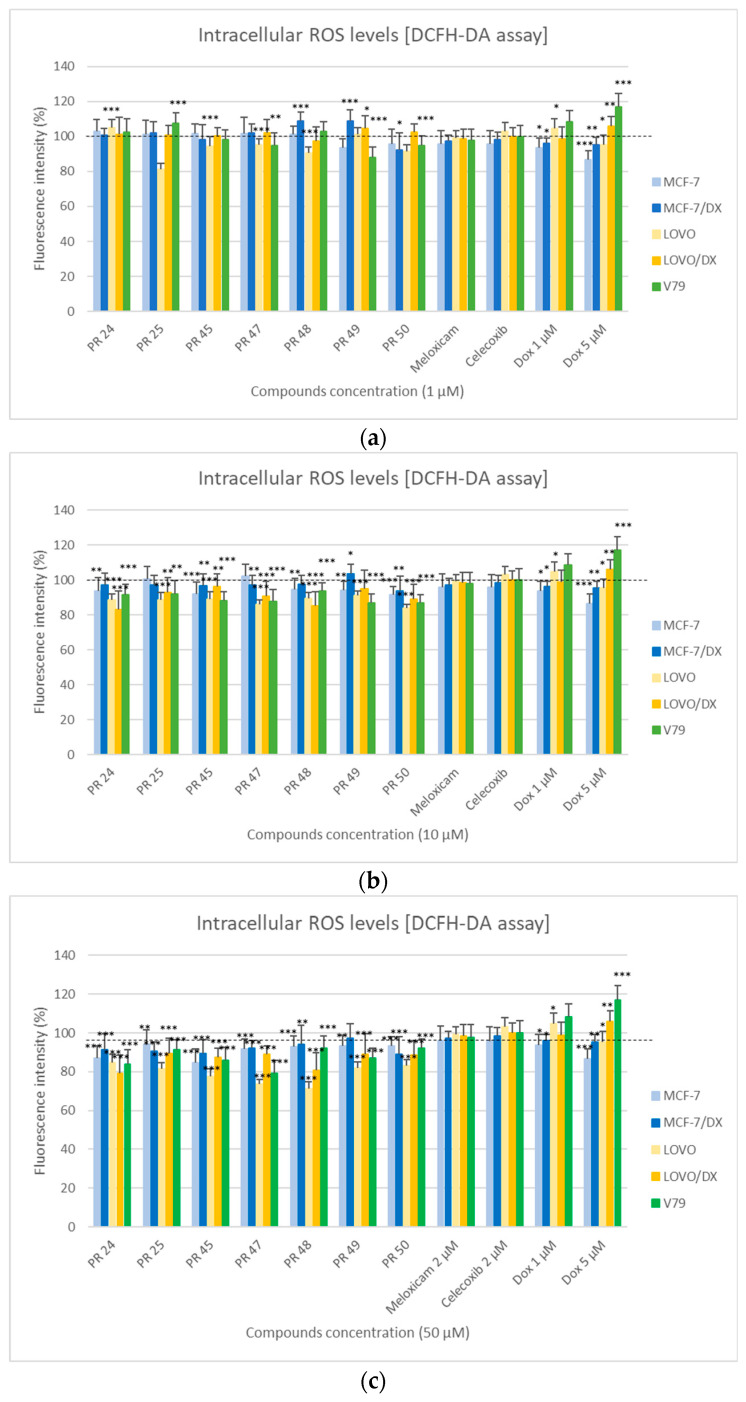
Effect of the tested compounds PR24–PR50 on the viability of cancer (MCF-7, MCF-7/DX, LOVO, LOVO/DX) and normal (V79) cells at a concentration of 1 µM (**a**), 10 µM (**b**) and 50 µM (**c**) evaluated by the DCFH-DA assay after 24 h of incubation. Results are presented as the percentage of cell viability relative to the untreated control (100%). The presence of statistical significance markers (* *p* ≤ 0.05; ** *p* < 0.01; *** *p* < 0.001) indicates significant differences compared to the control group.

In Figure 3, the results of the DCFH-DA assay are presented, determining the effect of the tested compounds PR24–PR50 on the level of reactive oxygen species (ROS) in cancer cells (MCF-7, MCF-7/DX, LOVO, LOVO/DX) and normal cells (V79) after 24 h of incubation at three concentrations: 1, 10, and 50 µM.

The analysis of the obtained results showed that the effect of the tested compounds on ROS levels depended on both the cell type and the applied concentration. In most cases, a decrease in fluorescence intensity was observed with increasing compound concentration, indicating a reduced level of reactive oxygen species and, consequently, the potential antioxidant activity of the tested compounds.

The strongest antioxidant effect was observed at a concentration of 50 µM (Figure 3c), particularly in the LOVO and LOVO/DX cell lines, where for compounds PR47 and PR48 a clear decrease in fluorescence intensity was noted compared to the control. In the LOVO/DX line, this effect was slightly weaker than in LOVO cells, which may confirm the lower sensitivity of the drug-resistant colorectal cancer line.

In breast cancer cell lines MCF-7 and MCF-7/DX, a moderate but statistically significant decrease in ROS levels was observed in response to most of the tested derivatives. The most pronounced antioxidant effect was observed for compounds at the highest concentration (50 µM), particularly for compound PR45. In the MCF-7/DX line (doxorubicin-resistant), for most of the tested compounds, a tendency toward a smaller decrease in fluorescence signal was observed compared to the sensitive line, which may confirm the lower susceptibility of the resistant line, similarly to the LOVO/DX line.

In the case of normal V79 cells, the response to the tested derivatives was varied. At the highest concentration (50 µM), most of the tested compounds caused a decrease in ROS levels compared to the control. The most pronounced decrease in fluorescence intensity was observed for compound PR47. This effect may indicate the antioxidant activity of all the tested compounds also in normal cells.

At lower concentrations 1 and 10 µM (Figure 3a,b), smaller but statistically significant changes in ROS levels were observed compared to the control. In some cases (particularly for PR48 and PR49 in MCF-7/DX and PR49 in LOVO/DX), a statistically significant increase in fluorescence was observed, indicating that these compounds induce oxidative stress at lower concentrations.

#### 3.2.5. Rhodamine 123 Assay

**Figure 4:**
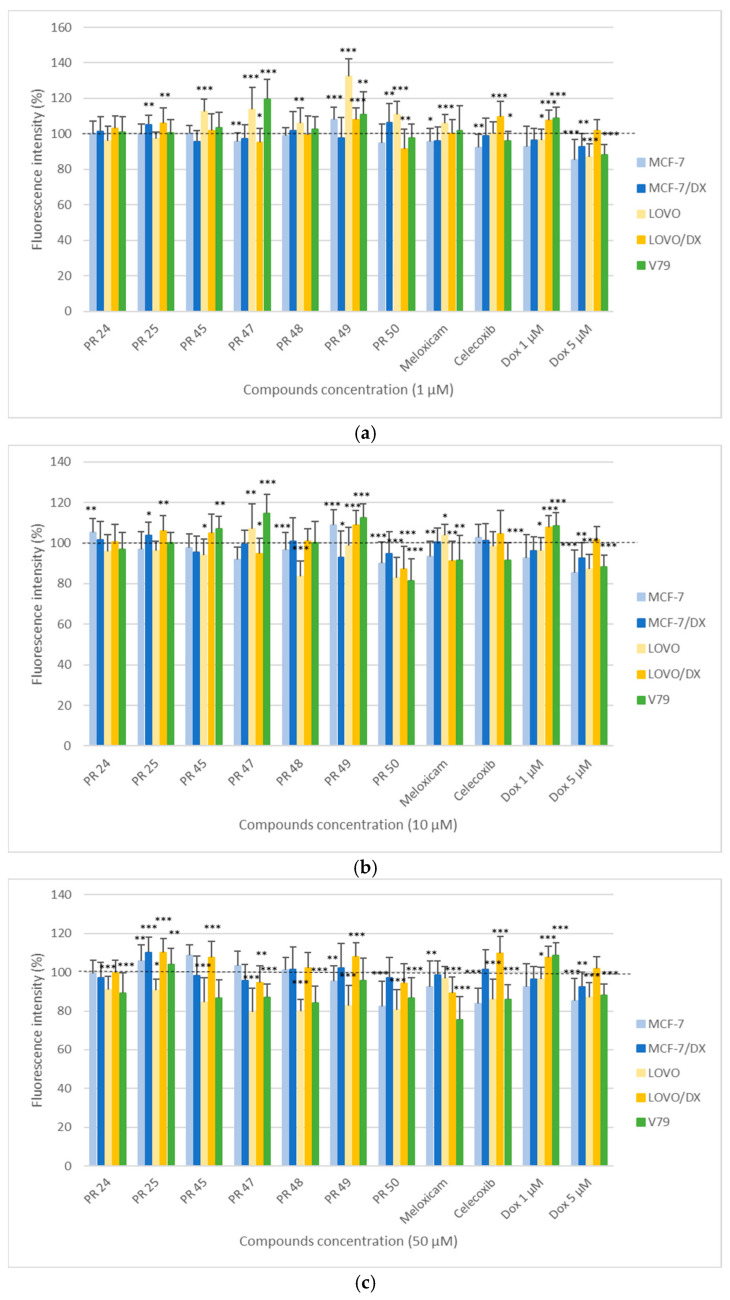
Effect of the tested compounds PR24–PR50 on the viability of cancer (MCF-7, MCF-7/DX, LOVO, LOVO/DX) and normal (V79) cells at a concentration of 1 µM (**a**), 10 µM (**b**) and 50 µM (**c**) evaluated by the Rhodamine 123 assay after 24 h of incubation. Results are presented as the percentage of cell viability relative to the untreated control (100%). The presence of statistical significance markers (* *p* ≤ 0.05; ** *p* < 0.01; *** *p* < 0.001) indicates significant differences compared to the control group.

In Figure 4, the results of the Rhodamine 123 (Rh123) assay are presented, determining the effect of the tested compounds PR24–PR50 on the mitochondrial membrane potential in cancer cells (MCF-7, MCF-7/DX, LOVO, LOVO/DX) and normal cells (V79) after 24 h of incubation at three concentrations: 1, 10, and 50 µM.

The obtained results indicate that the effect of the tested derivatives on the mitochondrial membrane potential depended on both the type of cells and the concentration of the compound. In most cases, a decrease in Rhodamine 123 fluorescence intensity was observed with increasing concentration, which indicates a reduction in mitochondrial membrane potential and disruption of mitochondrial function, potentially activating processes leading to apoptosis.

The strongest decrease in fluorescence signal was observed at the concentration of 50 µM (Figure 4c), particularly in the LOVO sublines, where all compounds caused a clearly significant reduction in fluorescence intensity compared to the control. In drug-resistant colorectal cancer cells (LOVO/DX), this effect was slightly weaker for compound PR47 and PR50 while for the other tested compounds the fluorescence intensity increased, which may result from greater stability of mitochondrial membranes in doxorubicin-resistant cells.

In breast cancer cell lines MCF-7 and MCF-7/DX, no reduction in mitochondrial potential was observed after incubation with most of the tested derivatives, while a moderate decrease in fluorescence intensity was noted for PR50 in sensitive MCF-7 subline at various concentrations. The smaller decrease in mitochondrial potential in resistant cells compared to the sensitive line may indicate the activation of compensatory mechanisms associated with increased resistance to stress factors.

In normal V79 cells, a decrease in fluorescence intensity was observed at the highest concentration (50 µM) for compounds PR45, PR47, and PR48, as well as at 10 µM for PR50. This suggests that most of the analyzed compounds within the tested concentration range did not cause significant disturbances in the mitochondrial membrane potential of normal cells.

At lower concentrations of 1 and 10 µM (Figure 4a,b), changes in mitochondrial membrane potential were less pronounced, although in some cases (PR50 at 10 µM) a statistically significant decrease in fluorescence intensity was still observed.

Considering the results of the Rhodamine 123 assay, it can be concluded that the tested compounds act most effectively on sensitive colorectal cancer cells subline (LOVO), reducing their mitochondrial membrane potential. This may result from mitochondrial damage and activation of apoptosis-related processes, as confirmed by the results of the cell viability assay (MTT). It also indicates the presence of cellular stress.

#### 3.2.6. Reference Compounds

In the study, reference compounds—meloxicam, celecoxib, and doxorubicin—were also used to compare their effects with the tested derivatives PR24–PR50. Both meloxicam and celecoxib did not cause significant changes in cell viability in most of the tested cell lines in the MTT assay, nor in fluorescence levels in the DCFH-DA assay, indicating the absence of a distinct cytotoxic effect at the applied concentrations (2 µM).

The IC_50_ values for meloxicam reported in the literature indicate its low cytotoxic activity. In studies conducted on MCF-7 cells, IC_50_ values exceeded 1.4 mM, confirming the very weak anticancer effect of this drug [63]. Similar results were obtained for C32 melanoma cells, where IC_50_ values were above 100 µM [64].

For celecoxib, the IC_50_ values reported in the literature were markedly lower but still indicative of moderate cytotoxicity. In colorectal cancer HT-29 cells, the IC_50_ value was approximately 30.4 µM [65]. These data confirm that celecoxib shows cytotoxic activity comparable to that of most of the tested derivatives PR24–PR50.

In contrast, doxorubicin exhibited high cytotoxic activity, as confirmed by both literature data and experimental results. In the present study, doxorubicin caused a significant decrease in cell viability in most of the examined cell lines in the MTT assay, both in concentrations of 1 and 5 µM.

The obtained results confirm that meloxicam and celecoxib are characterized by low cytotoxic potential, whereas doxorubicin, as expected, shows strong cytotoxic activity, confirming the accuracy of the applied methodology and the reliability of the results obtained for the tested derivatives PR24–PR50.

### 3.3. Molecular Docking Studies

**Figure 5:**
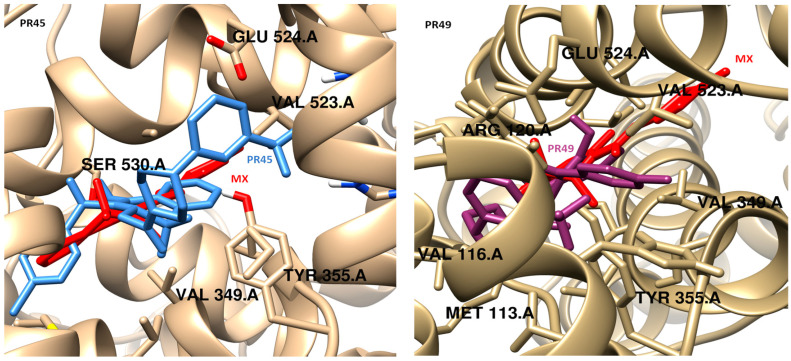
Comparison of the docking poses of PR45 (**left**, light blue) and PR49 (**right**, purple) with the reference inhibitor meloxicam (MX, red) within the COX-2 active site.

**Figure 6:**
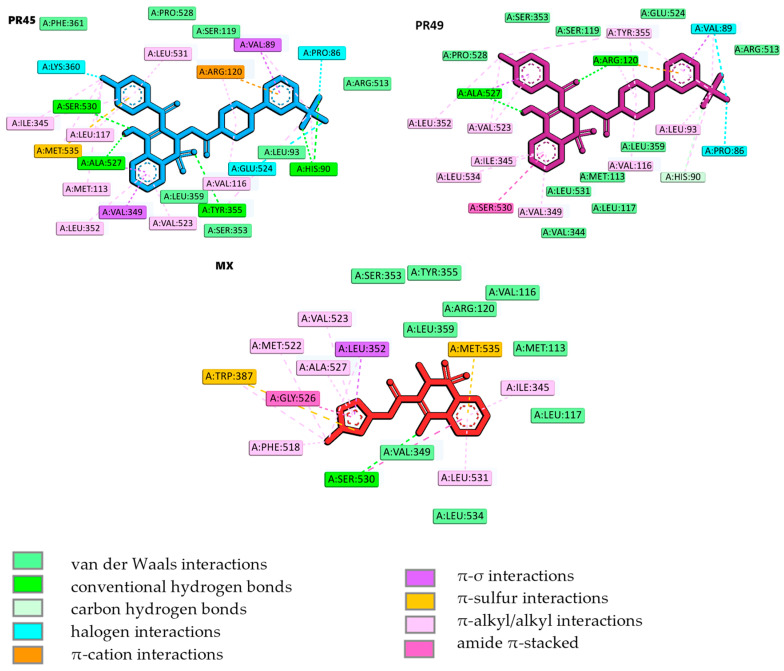
Intermolecular interactions of compounds PR45, PR49, and meloxicam (MX) in the binding cavity of COX-2.

The binding modes were determined by using molecular docking. The obtained data are presented in Figure 5 and Figure 6. Compound PR45, which exhibited best selectivity index COX-2/COX-1, and compound PR49, which was active exclusively towards COX-2, were selected for detailed analysis of their binding mode within the COX-2 binding site. Docking protocol validation was performed by re-docking the co-crystalized ligand (meloxicam) into the crystal structure of COX-2 binding site using the same docking parameters as those applied for the designed compounds. The accuracy of the docking protocol was evaluated by calculating the root mean square deviation (RMSD) between the docked pose and the crystallographic conformation. The obtained RMSD value below 1 Å, calculated using the LigRMSD web server, confirmed the reliability of the docking procedure [66]. Moreover, the docking protocol employed in this study has been previously validated and successfully applied in earlier work [67]. The binding score obtained for PR45 and PR49 were −10.83 and −10.00 kcal/mol, respectively. For comparison, the reference inhibitor meloxicam exhibited a docking score of −9.90 kcal/mol. These results indicate comparable predicted affinities towards the COX-2 active site, with PR45 showing negligibly stronger binding than both PR49 and meloxicam. It should be noted, however, that docking scores obtained using empirical scoring functions provide only semi-quantitative estimates of binding affinity and should be interpreted primarily in a comparative rather than an absolute manner.

As illustrated in Figure 5 and Figure 6, PR45 binds within the COX-2 active site like meloxicam, occupying the main catalytic channel and extending into the selectivity side pocket [60]. While meloxicam predominantly occupies the main catalytic channel. The observed selectivity of COX-2 inhibitors originates from subtle structural modifications within the enzyme binding site. Of particular importance is an additional selectivity pocket that contains Leu352, Ser353, Tyr355, Phe518, and Val523. This structural feature, resulting from the substitution of Ile523 with Val523 and the reorientation of the side chain of Tyr355, is consistently employed in the development of selective COX-2 inhibitors. In contrast, PR49 adopts a distinct binding orientation, which differs from that of both PR45 and meloxicam. Instead of penetrating deeply into the COX-2 selectivity pocket, PR49 is oriented towards an alternative region and extends in the opposite region to meloxicam. This altered binding mode may be related to structural differences between the two compounds, as PR49 has a methyl substituent instead of the chlorine atom present in PR45. The presence of the bulkier and more flexible CH_3_ group may impose steric constraints that favor an alternative accommodation within the binding cavity, thereby influencing the overall orientation of PR49. Designed compounds form key hydrogen bonds with Ser530 (PR45) and Arg120 (PR49). While interactions with Arg120 contribute to the general anchoring of the ligands within the catalytic domain and are also observed for meloxicam, they do not determine the selectivity of the compounds. In contrast to meloxicam the elongated aromatic substituents of PR45 penetrate deeper into the COX-2 selectivity side pocket, resulting in additional stabilizing interactions. This region is absent in COX-1 due to the presence of the previously mentioned bulkier residuum of Ile523. These fragments establish additional π-alkyl/alkyl interactions, van der Waals contacts and hydrogen bonds with His90, Leu93, Arg,513, Val523, and Met535, which are absent or less pronounced in the meloxicam-COX-2 complex.

Compound PR45 forms numerous nonpolar interactions that contribute to its high binding affinity. The trifluoromethylphenyl moiety is stabilized by π-cation, π-σ and halogen interactions with Arg120, Val89 and Pro86 amino acid residues, respectively. The 1,2-benzothiazine scaffold forms two hydrogen bonds with Ala527 and Ser530. Meanwhile, the chlorobenzoyl substituent is stabilized by numerous interactions with Met535 (π-sulfur), Lys350 (halogen), Ile345, Leu117, Leu531 (alkyl/π-alkyl) amino acid residues. In comparison, meloxicam forms fewer stabilizing contacts within the COX-2 selectivity pocket and does not engage the same extent of π-type and halogen interactions observed for PR45. Compound PR49 is anchored by hydrogen bonding interactions with Arg120, and Ala527. Further stabilization is achieved through halogen interactions with Pro86 and Val89. The occupancy of the pocket is increased by the van der Waals interactions of the 1,2-benzothiazine fragment with Met113, Leu117, Val344, Ser353, Leu359, and Leu531. The alternative orientation leads to less pronounced occupation of the canonical selectivity pocket, but enables PR49 to establish a complementary interaction network along the periphery of the COX-2 binding site. Molecular docking results indicate that both compounds are potent COX-2 inhibitors.

## 4. Discussion

Cytotoxicity studies demonstrated that all tested compounds reduced cancer cell viability in a cell line-dependent manner, with the most pronounced effects observed in the LOVO and LOVO/DX colon cancer cell lines. This observation is consistent with previous reports indicating that nonsteroidal anti-inflammatory drugs (NSAIDs) exhibit particularly strong anticancer activity in colorectal cancer models [68,69,70,71,72,73,74,75,76,77,78,79,80]. For instance, celecoxib, a selective COX-2 inhibitor, was shown to significantly reduce the number of duodenal polyps in patients with familial adenomatous polyposis (FAP) when administered at high doses (400 mg twice daily) [81]. The observed cytotoxic effects are further supported by the results of the Rhodamine 123 assay, which revealed a decrease in mitochondrial membrane potential in the same cell lines, indicating mitochondrial damage and activation of apoptosis-related mechanisms. Similar mitochondrial dysfunction and selective cytotoxicity toward colorectal cancer cells have been reported for other NSAIDs, suggesting that apoptosis-related pathways and mitochondrial impairment may contribute to their anticancer activity independently of cyclooxygenase inhibition [82].

Compounds PR48 and PR50 exhibited the highest cytotoxicity toward cancer cells; however, these compounds also showed substantial toxicity toward healthy cells, which limits their therapeutic potential. In contrast, PR24 and PR25 displayed low toxicity toward healthy cells but were also the least effective against cancer cells, resulting in no clear therapeutic advantage. Among the remaining compounds, PR45, PR47, and PR49 demonstrated a more favorable balance between anticancer activity and toxicity toward normal cells. Notably, PR45 and PR49 were the least toxic to healthy V79 cells, with estimated IC_50_ values of 123.0 µM and 178.0 µM, respectively, making them the most promising candidates within the tested series. Both compounds exhibited moderate cytotoxicity against cancer cell lines, with particularly strong activity against LOVO cells (IC_50_ = 12.3 and 17.5 µM, respectively) and their doxorubicin-resistant variant LOVO/DX (IC_50_ = 28.7 and 47.3 µM, respectively). Moreover, PR45 at a concentration of 1 µM inhibited LOVO/DX cell proliferation more effectively than the reference drug doxorubicin at the same concentration. Given the chemoresistant nature of the LOVO/DX cell line, this finding highlights the potential relevance of PR45 for further investigation.

Since overexpression of inducible cyclooxygenase-2 (COX-2), accompanied by increased production of prostaglandin E_2_ (PGE_2_), plays a key role in carcinogenesis, the ability of the oxicams derivatives to inhibit COX-2 was evaluated. The COX inhibition assay revealed that five out of seven tested compounds inhibited COX-2 activity, although their inhibitory potency was markedly lower than that of the reference compound celecoxib, indicating that COX-2 inhibition by the tested compounds is very weak. As expected for oxicam-derived structures, all tested compounds inhibited COX-1 to a greater extent than COX-2, reflecting a non-selective cyclooxygenase inhibition profile. Although the studied compounds retain the characteristic 1,2-benzothiazine core of oxicams, substantial structural modifications were introduced at the 2- and 3-positions of the thiazine ring. Specifically, the small methyl substituent at the 2-position was replaced with a bulky arylpiperazine moiety, while the arylcarboxamide group at the 3-position was substituted with a benzoyl group. These modifications likely altered the molecular geometry and steric properties of the compounds, limiting their optimal accommodation within the narrow COX active site and thereby reducing COX-2 inhibitory activity compared with classical oxicams.

Among the tested compounds, PR49 exhibited the strongest COX-2 inhibition, whereas PR45 showed the highest COX-2/COX-1 selectivity, justifying their selection for molecular docking studies. Docking analysis indicated that both compounds are capable of binding to the COX-2 active site with predicted affinities comparable to that of the reference drug meloxicam. It should be emphasized, however, that docking scores provide only semiquantitative estimates of binding affinity and cannot be directly translated into biological potency. Accordingly, the docking results were used to support structure–activity trends rather than to predict absolute inhibitory activity.

The COX inhibition data are further supported by membrane interaction studies, which demonstrated that all tested compounds were able to penetrate DMPC model membranes. This finding suggests that the compounds can access membrane-associated cyclooxygenase enzymes localized in the endoplasmic reticulum. Among the tested derivatives, PR49 exerted one of the strongest effects on the thermotropic properties of the phospholipid bilayer. This observation is consistent with previous studies indicating that the presence of an acetyl linker facilitates deeper penetration into phospholipid membranes and enhances their impact on membrane organization [42]. Moreover, earlier work demonstrated that oxicam derivatives containing an acetyl linker were effective modulators of multidrug resistance in LOVO/DX colon cancer cells [38].

From a structural perspective, PR45 and PR49 are closely related compounds. Both contain an acetyl linker connecting the thiazine and piperazine nitrogen atoms and an *m*-trifluoromethyl substituent on the phenylpiperazine moiety at the 2-position of the thiazine ring. The only structural difference between these compounds lies in the substituent at the 3-position of the thiazine ring: PR45 contains a *p*-chlorobenzoyl group, whereas PR49 bears a *p*-methylbenzoyl substituent. Structure–activity relationship (SAR) analysis indicated that the presence of an acetyl linker reduced the overall toxicity of the compounds, while the most toxic derivatives, PR48 and PR50, contained a propylene linker. Furthermore, the *p*-methylbenzoyl substituent in PR49 appeared to enhance COX-2 selectivity, whereas the *p*-chlorobenzoyl group in PR45 increased cytotoxicity, particularly against the LOVO/DX cell line. In summary, the structural modifications introduced into the oxicams scaffold resulted in reduced COX-2 inhibitory activity but enhanced cytotoxicity toward both cancerous and healthy cells. Importantly, certain substituents were found to reduce toxicity toward healthy cells (e.g., the acetyl linker between the thiazine and piperazine nitrogen atoms) while increasing cytotoxicity toward cancer cells (e.g., chlorine or bromine substituents and the *m*-trifluoromethyl group).

## 5. Conclusions

Although the compounds evaluated in this study exhibit lower COX inhibitory activity than clinically available agents and most do not demonstrate superior cytotoxic potency compared to established drugs, the results provide a rational basis for further structural optimization. Taking into account all studies conducted, compounds PR45 and PR49 appear to be the most promising candidates for further investigation. Both compounds exhibited significant cytotoxic activity against the LOVO and LOVO/DX cell lines while showing considerably lower toxicity toward healthy cells. Notably, PR45 at a concentration of 1 µM inhibited LOVO/DX cell proliferation more effectively than the reference compound doxorubicin at the same concentration. PR45 demonstrated the highest COX-2/COX-1 selectivity, whereas PR49 showed the strongest COX-2 inhibitory activity. The dual activity of these compounds, combining COX inhibition and cytotoxic effects, may be particularly relevant for cancer chemoprevention, especially in inflammation-associated malignancies such as colorectal cancer, where COX-2 is a key therapeutic target and may contribute to disease prevention and progression control. However, COX-2 inhibition is weak and unlikely to be the primary mechanism underlying the antitumor effects of these compounds. These limitations should be explicitly taken into account when interpreting their potential applications. Overall, both PR45 and PR49 exhibit promising pharmacological potential; nevertheless, further mechanistic studies are required to clarify their precise mode of action and evaluate their suitability for future therapeutic development.

## Figures and Tables

**Table 1:** Chemical structures of studied oxicam derivatives. For synthesis route and experimental data please see [42,43].

Compound Abbreviation	Molecular Weight [g/mol]	Chemical Structure
**PR24**	535.63	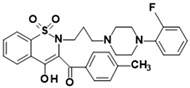
**PR25**	535.59	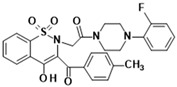
**PR45**	606.01	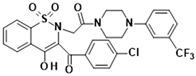
**PR47**	650.46	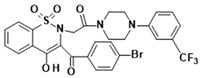
**PR48**	650.51	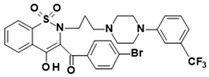
**PR49**	585.59	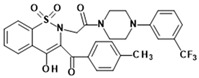
**PR50**	585.64	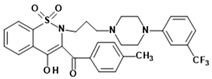

**Table 2:** Estimated IC_50_ values determined by the MTT assay.

Compound	MCF-7	MCF-7/DX	LOVO	LOVO/DX	V79
	[µM]				
**PR24**	nd	nd	nd	nd	108.0
**PR25**	nd	nd	nd	110.0	nd
**PR45**	nd	54.4	12.3	28.7	123.0
**PR47**	nd	87.4	11.0	28.5	55.6
**PR48**	nd	15.3	4.7	54.2	13.7
**PR49**	nd	nd	17.5	47.3	178.0
**PR50**	nd	18.3	8.8	nd	16.2

nd (not determined) indicates that IC_50_ value could not be determined within the tested concentration range.

**Table 3:** IC_50_ values estimated for COX-1 and COX-2 activities at 2 min of incubation with the tested compounds [mean; SD].

Compound	Cyclooxygenase Inhibition Assay IC_50_ [µM]		SI IC_50_(COX-2)/ IC_50_(COX-1)
	COX-1	COX-2	
**PR24**	115.50 (9.6)	359.80 (58.2)	3.10
**PR25**	nd	nd	
**PR45**	241.05 (81.6)	507.80 (72.1)	2.10
**PR47**	41.55 (9.05)	206.20 (31.6)	4.96
**PR48**	103.20 (10)	377.35 (22.1)	3.66
**PR49**	nd	87.50 (0.49)	
**PR50**	nd	nd	
celecoxib	6.53 (0.01)	0.72 (0.01)	0.11

nd (not determined) indicates that IC_50_ value could not be determined within the tested concentration range.

## Data Availability

The original contributions presented in this study are included in the article. Further inquiries can be directed to the corresponding authors.

## Abbreviations

The following abbreviations are used in this manuscript:

COX	cyclooxygenase
COX-1	cyclooxygenase 1
COX-2	cyclooxygenase 2
DCFH-DA	2′,7′-dichlorodihydrofluorescein diacetate
DMPC	1,2-dimyristoyl-*sn*-glycero-3-phosphocholine
DMSO	dimethyl sulfoxide
DPPC	dipalmitoylphosphatidylcholine
DSC	differential scanning calorimetry
IC_50_	half-maximal inhibitory concentration
LOVO	colorectal cancer cell line
LOVO/DX	doxorubicin-resistant colorectal cancer cell subline
MCF-7	breast cancer cell line
MCF-7/DX	doxorubicin-resistant breast cancer cell subline
MTT	3-(4,5-dimethylthiazol-2-yl)-2,5-diphenyltetrazolium bromide
MX	meloxicam
NSAIDs	nonsteroidal anti-inflammatory drugs
Rh123	Rhodamine 123
RNS	reactive nitrogen species
ROS	reactive oxygen species
SI	selectivity index
TMPD	*N,N,N′,N′*-tetramethyl-*p*-phenylenediamine
V79	normal cell line (fibroblast)
